# Middle Holocene daily light cycle reconstructed from the strontium/calcium ratios of a fossil giant clam shell

**DOI:** 10.1038/srep08734

**Published:** 2015-03-04

**Authors:** Masako Hori, Yuji Sano, Akizumi Ishida, Naoto Takahata, Kotaro Shirai, Tsuyoshi Watanabe

**Affiliations:** 1 Atmosphere and Ocean Research Institute, The University of Tokyo, Chiba, Japan; 2 Department of Earth and Planetary Sciences, Hokkaido University, Sapporo, Japan

## Abstract

Insolation is an important component of meteorological data because solar energy is the primary and direct driver of weather and climate. Previous analyses of cultivated giant clam shells revealed diurnal variation in the Sr/Ca ratio, which might reflect the influence of the daily light cycle. We applied proxy method to sample from prehistoric era, a fossil giant clam shell collected at Ishigaki Island in southern Japan. The specimen was alive during the middle Holocene and thus exposed to the warmest climate after the last glacial period. This bivalve species is known to form a growth line each day, as confirmed by the analysis of the Sr enrichment bands using EPMA and facilitated age-model. We analyzed the Sr/Ca, Mg/Ca and Ba/Ca ratios along the growth axis, measuring a 2-μm spot size at 2-μm interval using NanoSIMS. The Sr/Ca ratios in the winter layers are characterized by a striking diurnal cycle consisting of narrow growth lines with high Sr/Ca ratios and broad growth bands with low Sr/Ca ratios. These variations, which are consistent with those of the cultivated clam shell, indicate the potential for the reconstruction of the variation in solar insolation during the middle Holocene at a multi-hourly resolution.

In paleoclimatic studies, valuable information, such as historical seawater temperatures^1^, salinity^2^, pH^3^, and nutrient availability^4^, has been derived experimentally from analyses of the stable isotopes and of the trace element concentrations of marine calcium carbonates, such as coral skeletons and foraminifera tests. Insolation is a direct consequence of solar energy and a driving force of environmental change^5^. However, the effect of increasing solar energy is not uniform, resulting in heavy rain or serious drought depending on the locality. Attempts to develop a proxy for insolation^6^ have included the use of carbonate samples, but these attempts have not been successful. This lack of success is partially attributable to the difficulty in distinguishing the variation in insolation from that of temperature by the analyses conducted at the scale of 100s of μm^7^ because solar energy is the driver of both meteorological parameters. Therefore, the insolation and temperature are expected to change following a similar pattern. A recent study has reported that the Sr/Ca ratios in cultivated giant clam shells exhibit striking diurnal variation, reflecting the daily light cycle^8^.

For this study, we applied a proxy method to a fossil giant clam (*Tridacna gigas*) shell to demonstrate the potential of the use of the Sr/Ca ratio as a proxy for paleo-insolation. The specimen was collected at the Shiraho Coast of Ishigaki Island in the southwestern portion of the Ryukyu Archipelago, southern Japan (Supplementary Fig. S1), and its living age was determined as the middle Holocene using a radiocarbon method (Fig. 1a). The results were compared with those of modern specimens, which were cultivated at the same island^8^.

## Results

**Figure 1:**
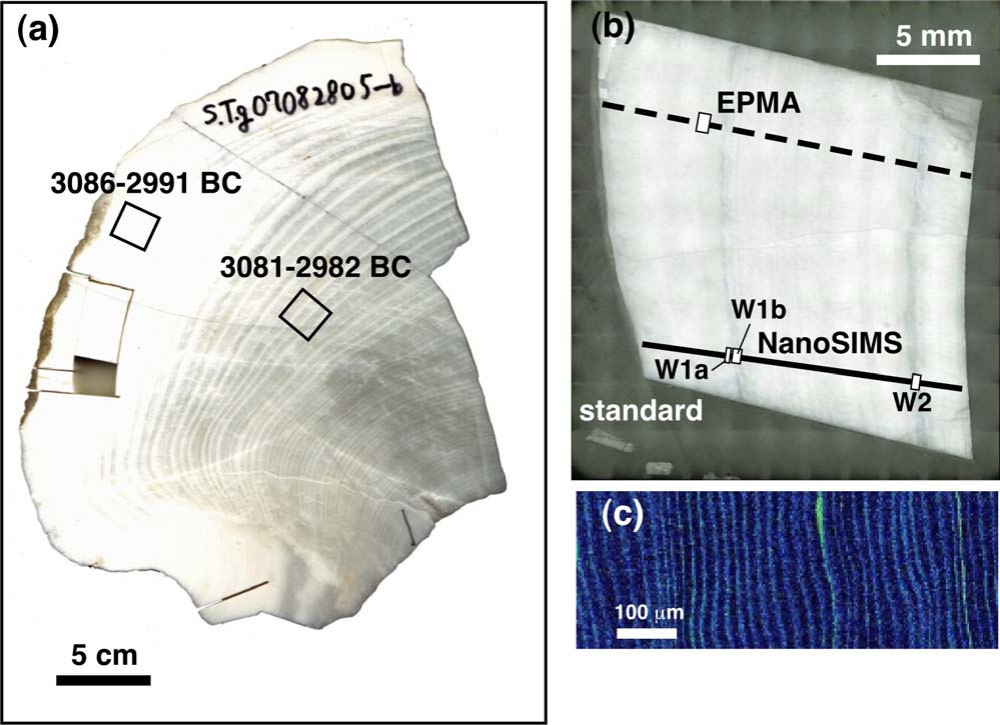
(a) Whole section of the analyzed fossil giant clam shell. The individual radiocarbon ages are given for the two samples (portions of the outer and middle layers). (b) The portion of the shell outer layer mounted in an Araldite resin disk, together with a carbonate standard. The dotted and solid lines, respectively, portray the results of EPMA and NanoSIMS analyses along the growth axis. W1a, W1b and W2 represent sections of the “high-resolution analysis”. (c) Strontium concentration map of the sample enlarged from the square marked in (b).

**Figure 2:**
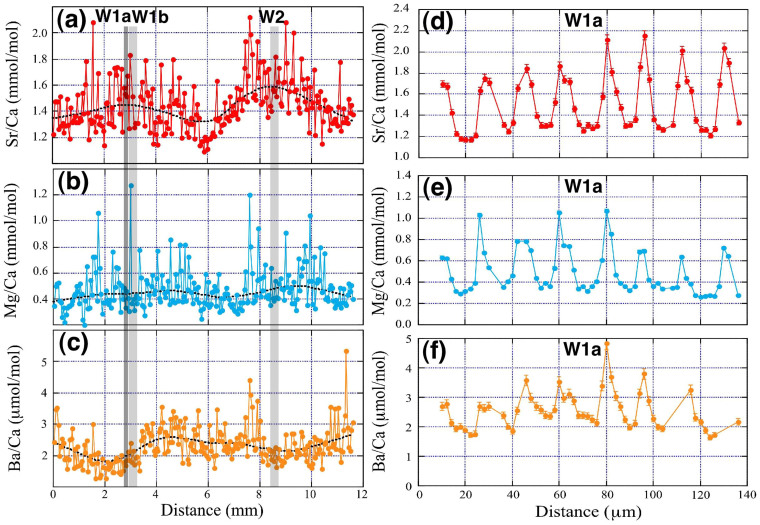
(a) Low-resolution analyses of the Sr/Ca ratio, (b) Mg/Ca ratio, and (c) Ba/Ca ratio along the growth axis of the clam shell, marked by the solid line in Fig. 1b. (d) High resolution analyses of Sr/Ca ratio, (e) Mg/Ca ratio, and (f) Ba/Ca ratio along the growth axis in the section marked by W1a in Fig. 1b.

Figure 2a presents a long dataset of Sr/Ca ratios, referred to herein as the “low-resolution analysis”. Measurements were conducted at a 50 μm resolution along the growth axis of the fossil shell from one edge to the other end using NanoSIMS (the solid line in Fig. 1b). The Sr/Ca ratios vary from 1.09 to 2.12 mmol/mol, with a mean of 1.44 ± 0.20 mmol/mol (hereafter, the error assigned to the mean value is 1σ standard deviation). In the low-resolution analysis, there are two apparent periodical variations in Sr/Ca ratios; the maximum values corresponds to the dark lines (Fig. 1b). Figures 2b and 2c, respectively, present the associated low-resolution Mg/Ca and Ba/Ca measurements. There is a weak positive correlation between the Mg/Ca and Sr/Ca ratio, whereas there is no obvious correlation between the Ba/Ca and Sr/Ca ratios.

The enlarged images of the Sr concentration maps of the fossil bivalve shell contain a cyclic pattern, alternating between high and low Sr contents for thin and thick bands, respectively (Fig. 1c). The period from one Sr/Ca maximum (that is the middle point of a dark and opaque area in Fig. 1b) to another contains approximately 350 Sr enrichment bands by EPMA. Figure 2d presents the Sr/Ca results for the “high-resolution analysis”. Measurements were collected at a 2 μm resolution along the growth axis within the dark and opaque area denoted as “W1a” in Fig. 1b, which corresponds to the region with the maximum Sr/Ca ratios in the low-resolution analysis. The Sr/Ca ratio varies from 1.17 to 2.16 mmol/mol, with a mean of 1.51 ± 0.27 mmol/mol. The relatively longer datasets of Sr/Ca ratios collected for sections “W1b” and “W2” in Fig. 1b exhibit similar cyclic variations to W1a (Supplementary Fig. S2), even though the data for section W2 are more irregular. Figures 2e and 2f, respectively, present the associated high-resolution Mg/Ca and Ba/Ca measurements at the position W1a of Fig. 1b. There are apparent periodical variations in the Mg/Ca and Ba/Ca ratios, with seven cycles contained within the section, which is consistent with the Sr/Ca variations, reflecting the clear positive correlation (Supplementary Fig. S3). The relatively longer datasets for the Mg/Ca ratios of sections “W1b” and “W2” in Fig. 1b (Supplementary Fig. S2) are characterized by similar periodical variations to those of W1a, whereas the Ba/Ca ratios of W2 follow no clear cyclic pattern.

Table 1 lists the basic statistics of the fossil clam shell chemistry data of this study (periods W1a, W1b and W2) together with those of the cultivated samples from the same island^8^. The growth rate of each period was calculated by multiplying the number of data points per day by the analytical interval of 2 μm. The first listed modern clam shell specimen is characterized by a growth rate of 21 ± 5 μm/day, which is almost double that of the other modern specimen, 10 ± 3 μm/day (Table 1). The growth rate of the Holocene fossil sample collected for this study varied from 12 ± 4 to 16 ± 3 μm/day, which is well within the range of the modern clam shells. The mean Sr/Ca ratio of the fossil clam was 1.51 ± 0.27 mmol/mol for the W1a period, 1.53 ± 0.29 mmol/mol for W1b, and 1.26 ± 0.14 mmol/mol for W2. The results for the first winter (W1a and W1b) indicate a similar level of variation as that observed for the modern, cultivated samples (1.54 ± 0.20 and 1.51 ± 0.26 mmol/mol), whereas the second winter (W2) is characterized by a relatively small Sr/Ca ratio and low level of variation.

## Discussion

Both the low- and high-resolution measurements of the Sr/Ca ratios of a fossil giant clam shell were characterized by cyclic patterns at varying time scales, similar to those observed for the cultivated clam shells from the same island^8^. The daily maximum and minimum Sr/Ca ratios, together with dynamic range of the fossil sample results, are consistent with those of the cultivated samples within the 1σ error, except for the second winter (Table 1). The similarity between the variations in the Sr/Ca ratios of the fossil and modern samples suggests that the same mechanisms control aragonite growth and the incorporation of these elements.

An important feature commonly observed from fossil and modern samples is the presence of daily bands with alternating high and low Sr/Ca ratios. In the W1a section, for example, one cycle has approximately eight or nine data points, where peaks generally consist of one or two data points, whereas the troughs consist of a greater number of data points (Fig. 2d). These variations are consistent with the Sr concentration map observed by EPMA (Fig. 1c). The wider bands of Sr depletion are likely to represent daytime because the clam shell calcification rate is expected to be enhanced by the photosynthetic activity of the symbiotic dinoflagellates^9^,^10^. By contrast, the narrower enrichment bands are assigned to nighttime. The autocorrelation analysis of the Sr/Ca data (Supplementary Fig. S4) suggests that the periodic variation over a 20 μm shell distance might represent the mean daily growth bandwidth. Thus, the 2 μm spatial resolution of the NanoSIMS analysis might capture a few hours of time.

The Sr enrichment bands, therefore, can be used for facilitating the age-model determination along the growth axis (dotted line in Fig. 1b). In our study, 350 micro-bands were observed between two maximum values of the Sr/Ca ratio in the low resolution analysis, which correspond to the middle portions of the narrow dark lines (Fig. 1b). Thus, the period between two narrow dark lines (with high Sr/Ca ratios) represents the annual carbonate precipitation. This conclusion is reasonable as suggested in the previous study^11^, which determined the shell growth rate based on the radioactivity from ^90^Sr deposited in the shell grown during the testing of nuclear weapons. According to the observations, it was also concluded that differentiation of the dark and light annual bands represent the seasonal variation; the dark and opaque areas were assigned to winter growth, whereas the lighter and more translucent areas were assigned to summer growth. The fossil sample analyzed for this study was characterized by a similar pattern: narrower dark lines and wider light bands (Fig. 1b). Therefore, it is inferred that the maximum Sr/Ca ratios in the narrow dark bands represent winter and that the minimum ratios in the wide light bands represent summer (Fig. 2a).

Here, we elucidate the driving mechanisms of the variability's in minor and trace elements over time. Two steps might build up the aragonite shell: first, the ionic transport of Ca^2+^ from ambient seawater to extrapallial fluid (EPF); and second, precipitation from EPF to the shell skeleton. The first step has three transport pathways through the epithelial mantle in a marine bivalve^12^,^13^,^14^: (1) the intercellular diffusive pathway, (2) the active pathway via Ca^2+^-ATPase pump, and (3) the major Ca^2+^-channel pathway (see Supplementary Fig. S5). The intercellular channel for Ca^2+^ ions might act similarly to those for Mg^2+^, Sr^2+^ and Ba^2+^ ions, whereas the Ca^2+^-ATPase pump and the Ca^2+^-channel might be selective for Ca^2+^ ions. The Ca^2+^-ATPase pump is activated during the daytime and produces high-calcification rates because it is induced by enzymes that are activated by solar energy^12^. Thus, the Sr/Ca ratio of EPF may be relatively higher at night. The Ca^2+^-channel hypothesis supports the model by which a reduction of Ca selectivity might occur at high calcification rates because the ion diffusive transport is driven by the Ca^2+^ gradient^13^. This type of response would result in a higher Sr/Ca ratio in the daytime, which is inconsistent with the observations. The lower solar radiation in winter and the non-insolation at night might produce higher Sr/Ca ratios in association with the reduction in the Ca^2+^-ATPase pump.

The coincident variations in the Mg/Ca and Ba/Ca ratios, particularly in the high-resolution analysis (Figs. 2d, 2e and 2f), are also explained by the transport-pathway hypothesis because Mg and Ba have markedly similar chemical characteristics. However, the characteristics of the low-resolution analysis differ in that the temporal variation in the Mg/Ca ratio is spiky relative to the Sr/Ca ratio. This larger variation is likely attributed to the stronger biological control over Mg, as suggested by the spatial variation in the aragonite skeleton of a reef-building coral^15^. The Ba/Ca ratio might be affected by reef nutrient contents^16^ or salinity^17^, which are influenced by the occasional occurrence of heavy precipitation from summer to late autumn.

In the second step of shell formation, elemental fractionation might be governed by the kinetic process of inorganic precipitation of aragonite under the assumption of a homogeneous EPF composition with time. A laboratory experiment^18^ reported that the Sr/Ca exchange coefficient of aragonite increases with an increase in the precipitation rate. Following this relationship, the Sr/Ca ratio would increase during the daytime when the aragonite growth rate is high. However, this result is not supported by our observations, which indicate the opposite relationship during the daytime.

**Figure 3:**
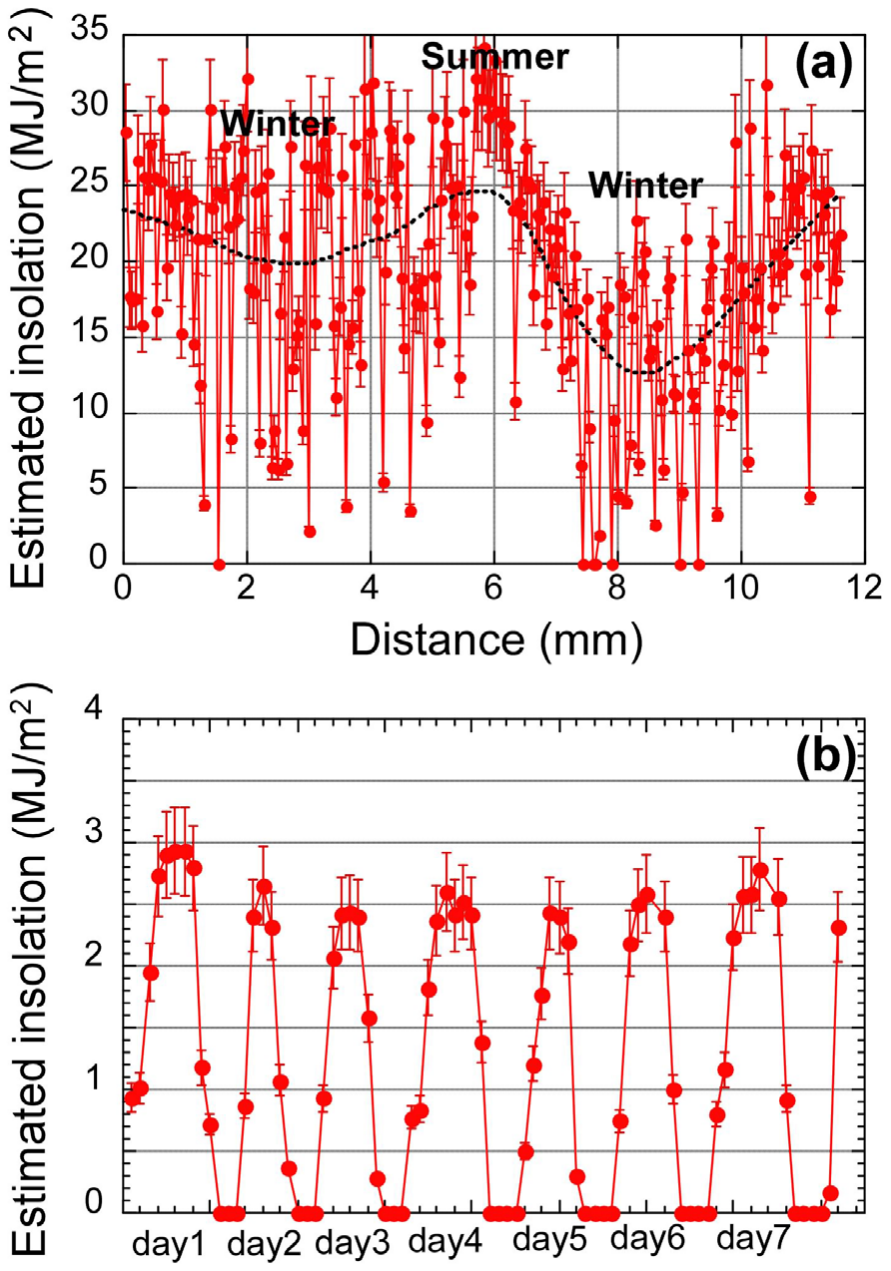
(a) Reconstructed daily insolation of the middle Holocene according to the low-resolution analysis of the Sr/Ca ratio in the fossil giant clam shell. The errors assigned to the symbols are those calculated using the Y-intercept (0.16) and inclination (0.009) from eq. (1) in quadrature. (b) Reconstructed hourly insolation of late winter according to the high-resolution analysis of the Sr/Ca ratio. The errors assigned to the symbols are again those calculated using the Y-intercept (0.17) and inclination (0.085) from equation (2) in quadrature.

According to the above discussion, we suggest that the Sr/Ca ratios of a fossil giant clam shell reflect the daily light cycle in the middle Holocene. Hence, we applied the empirical formula determined for the modern daily mean insolation and the corresponding monthly mean Sr/Ca ratio of cultivated giant clam shells^8^ to the current fossil clam shell:
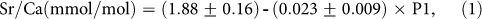
where P1 denotes the daily insolation (MJ/m^2^). Negative values are corrected to zero insolation (Fig. 3a). Note that this is the first reconstructed variation of daily insolation for approximately 5000 years ago. The weighted average of the hypothetical insolation with a 30% smoothing coefficient indicates a maximum daily value of 25 MJ/m^2^. This value is consistent with the mean of the present summer insolation on Ishigaki Island. By contrast, the minimum value of 19 MJ/m^2^ in the first winter (W1a and W1b) is greater than in the present winter, even though that of the second winter (W2) is a reasonable value of 12 MJ/m^2^. We have no plausible explanation for the high insolation in the first winter, but it might be related to the occurrence of the warmest climate after the last glacial period in the middle Holocene^19^.

In the same manner, the hourly insolation in winter in the middle Holocene was calculated using the equation previously developed for modern specimens^8^:
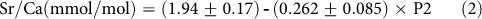
where P2 denotes the hourly insolation (MJ/m^2^). Here, we modified the Y-intercept to 1.94 ± 0.17, which is the mean peak Sr/Ca value of the fossil shell (Table 1), and this newly applied Y-intercept value agrees with the original value of 2.09 ± 0.49 for a modern specimen^8^ within the margin of error. The mean hourly peak insolation is 2.60 ± 0.17 MJ/m^2^ (Fig. 3b), which is comparable to the fair days in late winter for the previous ten years (2.70 ± 0.19 MJ/m^2^) on Ishigaki Island. Note that this is also the first reconstructed variation of the hourly insolation approximately 5000 years ago. We also calculated the variations in insolation for the longer datasets of “W1b” and “W2” (Supplementary Fig. S6). The first winter values (means of W1a and W1b) are significantly higher than those for the second winter (W2), which was also observed in the direct comparison of the cultivated and fossil specimens (see Table 1). This finding is consistent with the low-resolution analysis, and the high insolation in the first winter might be related to the warm climate during the middle Holocene^19^.

Our results characterize the hourly variation in insolation during the middle Holocene, as reconstructed from the Sr/Ca ratios of a fossil clam shell. We analyzed the minor and trace elements along the growth axis of a fossil giant clam shell collected from Ishigaki Island in southern Japan using NanoSIMS at a spatial resolution of 2 μm for a detailed analysis and 50 μm for the analysis of general trends. The former analysis is the finest spatial resolution available for studies of carbonate geochemistry. The age-model was facilitated by the analysis of the Sr enrichment bands with EPMA. A clear, daily light cycle was reconstructed for the Sr/Ca ratio for a subtropical region at a resolution of several hours for at approximately 5000 years ago. Further study might elucidate the influence of the warmest climate in the middle Holocene.

## Methods

We collected a fossil giant clam (*Tridacna gigas*) shell at the Shiraho Coast of Ishigaki Island (Supplementary Fig. S1). Shiraho is a well-developed fringing coral reef, similar to Kabira that is located on the same island where we cultivated giant clams for a previous study^8^.

One side of the fossil shell was cut. Then, a radial section was prepared using a dental cutting machine (Fig. 1a). Two powdered samples (portions of the outer and middle layers, Fig. 1a) were submitted to radiocarbon dating at the Accelerated Mass Spectrometry Center of Yamagata University. The two ages, calculated using the Marine09 calibration curve with a local reservoir effect (ΔR) of zero, were consistent, 3081–2982 BC and 3086–2991 BC (1σ). Another outer layer portion was cut and mounted in an Araldite resin disk together with a natural calcite ore standard^20^ (Fig. 1b). A mounted sample was polished with a polishing sheet with fine alumina grains embedded on the surface. The polished surface was coated with Pt–Pd vapor and examined using an electron probe micro-analyzer (EPMA JXA8900; JEOL, Tokyo, Japan) at the Atmosphere and Ocean Research Institute, The University of Tokyo.

After EPMA observation, the Pt–Pd coat was removed with ethanol and then coated again with gold to avoid charging during the ion probe analysis. Measurement of minor and trace elements was performed using a nanometer-scale secondary ion mass spectrometer (NanoSIMS NS50; Cameca SAS, Gennevilliers, France) installed at the Atmosphere and Ocean Research Institute, The University of Tokyo. We conducted measurements of two types: low-resolution and high-resolution analyses (Fig. 1b). For the “low-resolution” analysis, a 10 nA primary beam of ^16^O^−^ was focused on a 10 μm diameter spot with a 50 μm interval. For the “high-resolution” analysis, a 100 pA primary beam with a 2 μm spot was used at a 2 μm interval. Secondary ions were extracted by an accelerating voltage of 8 kV, and the ^24^Mg, ^44^Ca, ^88^Sr and ^138^Ba ions were measured simultaneously using a multi-ion counting system. Molecular interference was not observed, using a mass resolving power of 3100 at 10% peak height, except for the ^40^Ca_2_^26^Mg ^16^O_2_ on the ^138^Ba peak. The ^138^Ba/^44^Ca ratio was corrected by the following equation: (^138^Ba/^44^Ca)_cor_ = (^138^B/^44^Ca)_obs_ – (^24^Mg/^44^Ca)_obs_ × 0.000279; where “cor” and “obs”, respectively, signify the corrected and observed ratios^21^. All of the data were calibrated against a natural calcite standard from Mexico^20^, which is homogeneous for the Mg/Ca and Sr/Ca ratios. The errors associated with the Mg/Ca, Sr/Ca and Ba/Ca ratios are 2%, 1.5% and 5% at 1σ, respectively, as estimated by the repeated analysis of the standard.

## Author Contributions

M.H. performed all of the experimental analyses, including NanoSIMS and EPMA. Y.S. conceived the study and wrote the manuscript. M.H. and Y.S. prepared a revised version. A.I. and N.T. conducted the instrumental control of the NanoSIMS. K.S. and T.W. contributed to field sampling and advised on biomineral implications. All of the authors contributed to the interpretation of the results and to the discussion of the associated geochemistry of the fossil giant clam shell examined in this study.

## Supplementary Material

Supplementary Information Supplementary material

## Figures and Tables

**Table 1:** Basic statistics of clam shell chemistry data

Sample	Holocene fossil of this study			Modern cultivated*	
Species	*Tridacna gigas*			*Tridacna derasa*	
	W1a	W1b	W2	1	2
Season	first early winter	first late winter	second winter	From late-September to mid-October, 2005	From late-September to mid-October, 2005
**Number of data per day**	8.0 ± 1.4	6.2 ± 1.8	7.9 ± 1.4	10.4 ± 2.5	5.1 ± 1.4
**Growth rate (μm/day)**	16 ± 3	12 ± 4	16 ± 3	21 ± 5	10 ± 3
**Mean Sr/Ca (mmol/mol)**	1.51 ± 0.27	1.53 ± 0.29	1.26 ± 0.14	1.54 ± 0.20	1.51 ± 0.26
**Maximum Sr/Ca (mmol/mol)**	1.94 ± 0.17	1.99 ± 0.19	1.46 ± 0.07	1.87 ± 0.10	1.87 ± 0.16
**Minimum Sr/Ca (mmol/mol)**	1.25 ± 0.05	1.29 ± 0.13	1.10 ± 0.05	1.32 ± 0.05	1.26 ± 0.08
**Dynamic range (mmol/mol)**	0.69 ± 0.18	0.70 ± 0.23	0.36 ± 0.09	0.55 ± 0.11	0.61 ± 0.18

Error values represent the standard deviation at 1σ.

*: Original data are from Sano et al. [8].
