# Supplementary material for: Middle Holocene daily light cycle reconstructed from the strontium/calcium ratios of a fossil giant clam shell
Source: Sci Rep. 2015 Mar 4;5:8734. doi: 10.1038/srep08734 (PMC4348650; doi:10.1038/srep08734)
Supplement: Supplementary Information — Supplementary material [file srep08734-s1.pdf]

Supplementary Material

Middle Holocene daily light cycle reconstructed from the strontium/calcium ratios of a fossil giant clam shell

Masako Hori[1], Yuji Sano[1]*, Akizumi Ishida[1], Naoto Takahata[1], Kotaro Shirai[1], and Tsuyoshi Watanabe[2]

[1]Atmosphere and Ocean Research Institute, The University of Tokyo, Chiba, Japan

[2]Department of Earth and Planetary Sciences, Hokkaido University, Sapporo, Japan

*Correspondence and requests for materials should be addressed to Y.S. (e-mail: ysano@aori.u-tokyo.ac.jp)

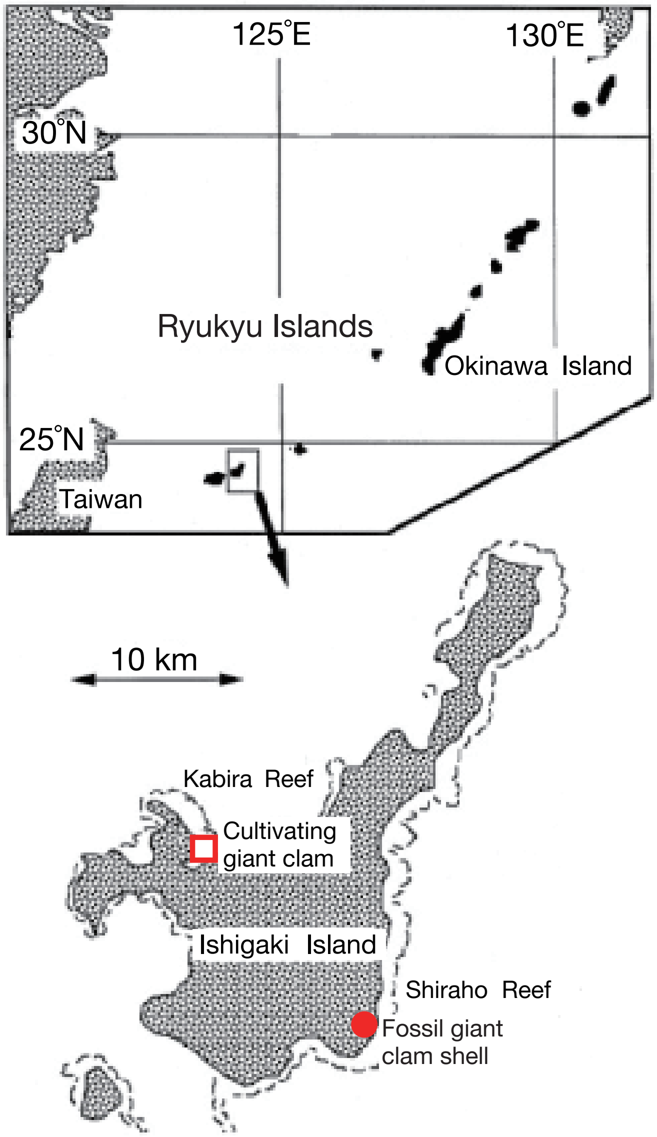

Supplementary Figure S1. (a) Location of Ishigaki Island in the southwestern part of the Ryukyu Archipelago, southern Japan. (b) Sampling site of the fossil giant clam shell in the Ishigaki Island together with that of cultivating giant clam in previous work[1].

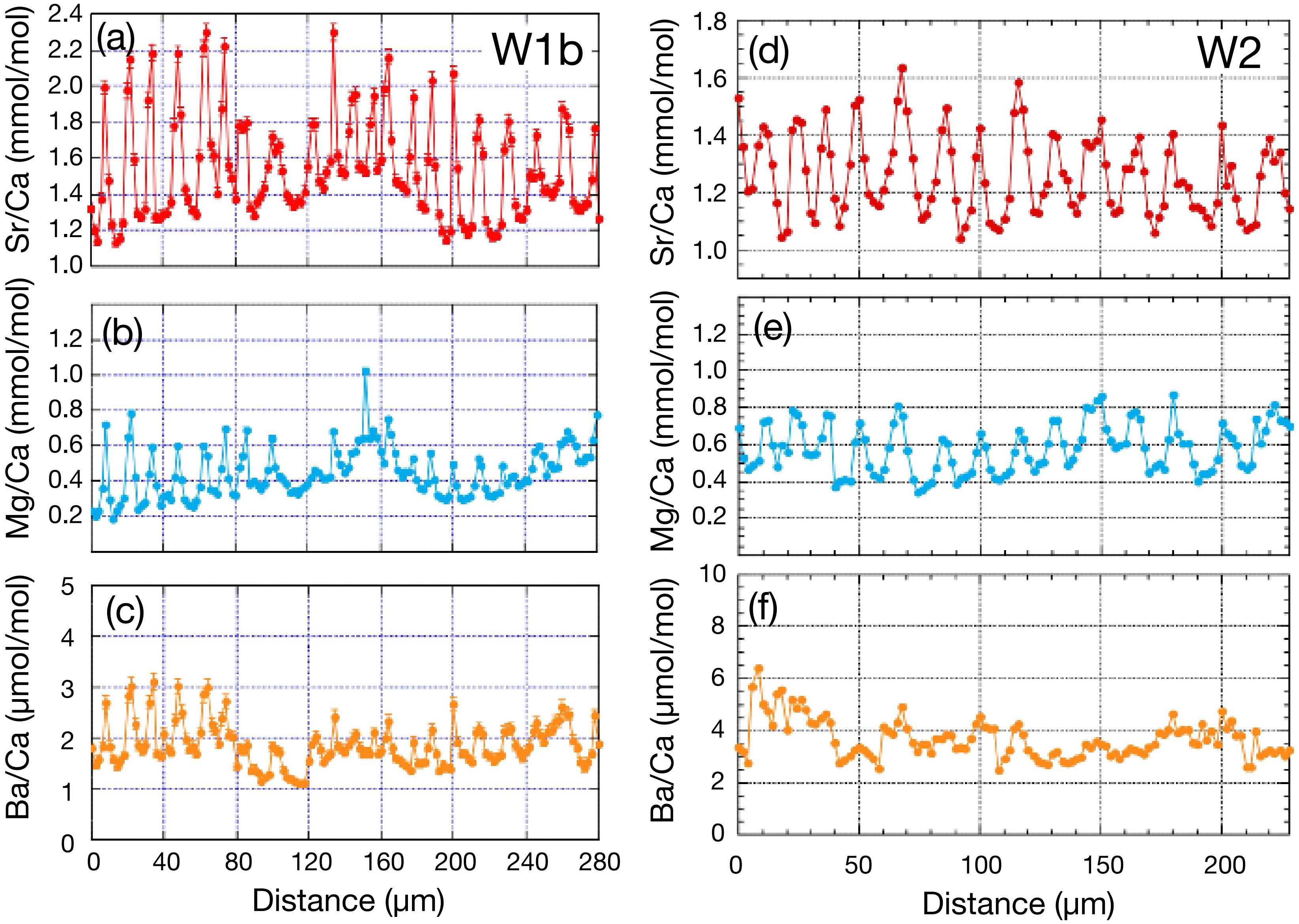

Supplementary Figure S2. (a) High resolution analyses of Sr/Ca ratio, (b) Mg/Ca ratio and (c) Ba/Ca ratio along the growth axis in the section marked by W1b in Fig. 1b. (d) Those of Sr/Ca ratio, (e) Mg/Ca ratio, and (f) Ba/Ca ratio of W2 in Fig. 1b.

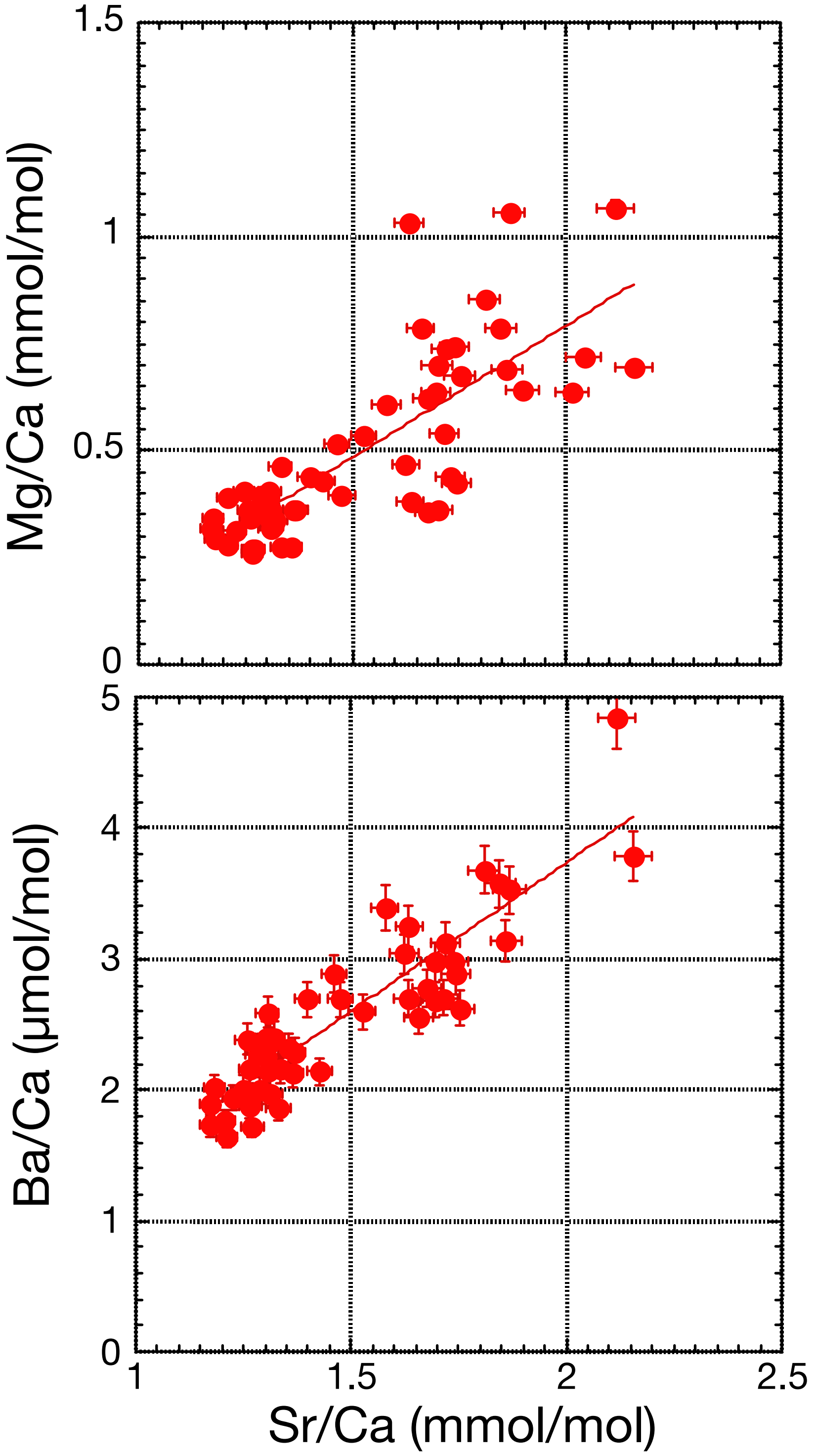

Supplementary Figure S3. (a) A correlation diagram between Sr/Ca ratio and Mg/Ca ratio of high resolution analysis of section W1a in Fig. 1b. (b) That between Sr/Ca ratio and Ba/Ca ratio. Error assigned to the symbol is one sigma.

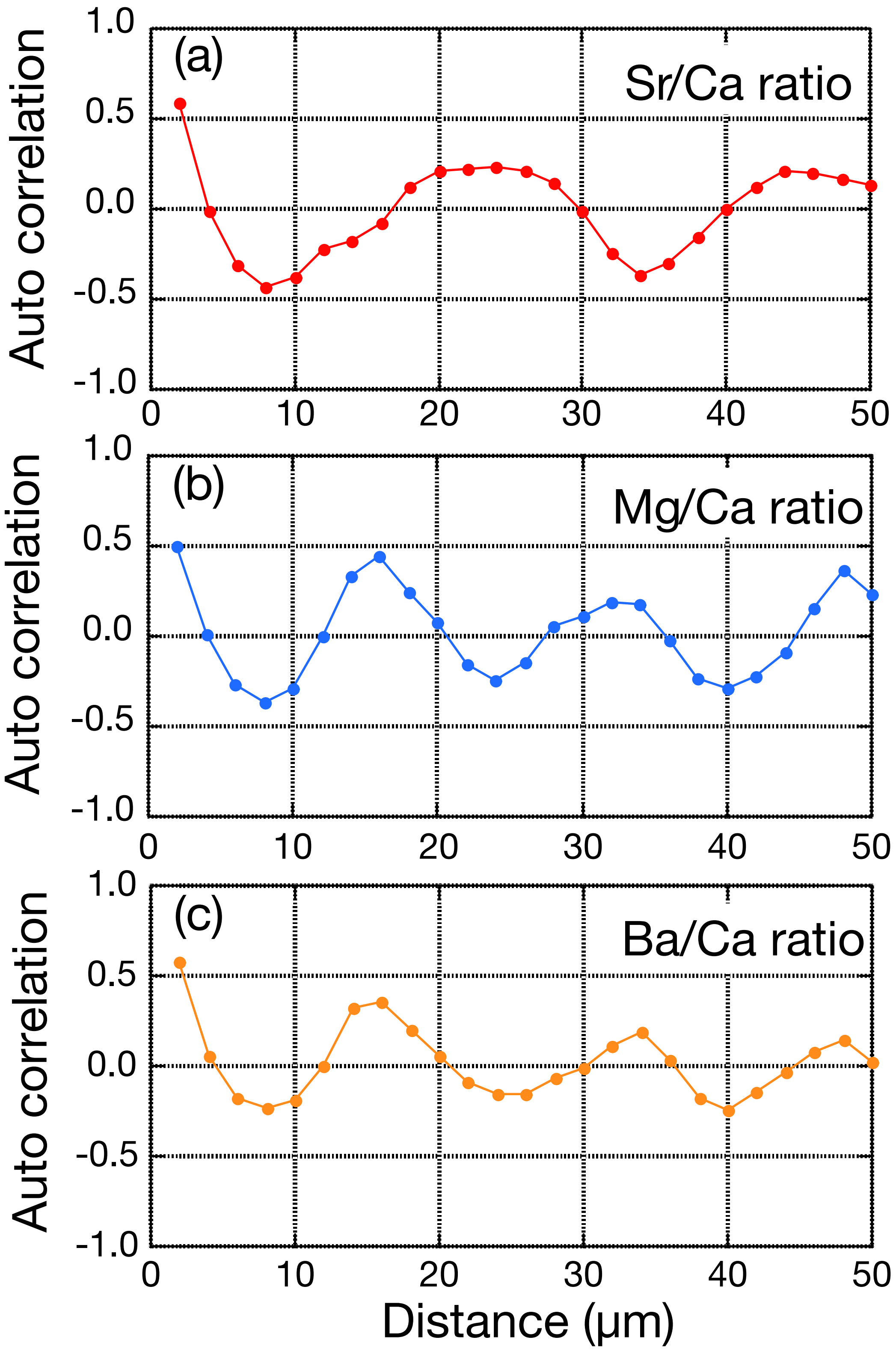

Supplementary Figure S4. (a) Auto correlation analyses of high resolution Sr/Ca data, (b) Mg/Ca data and (c) Ba/Ca data of Figs. 2d, 2e and 2f, respectively. Horizontal axis is a distance unit of micro meter.

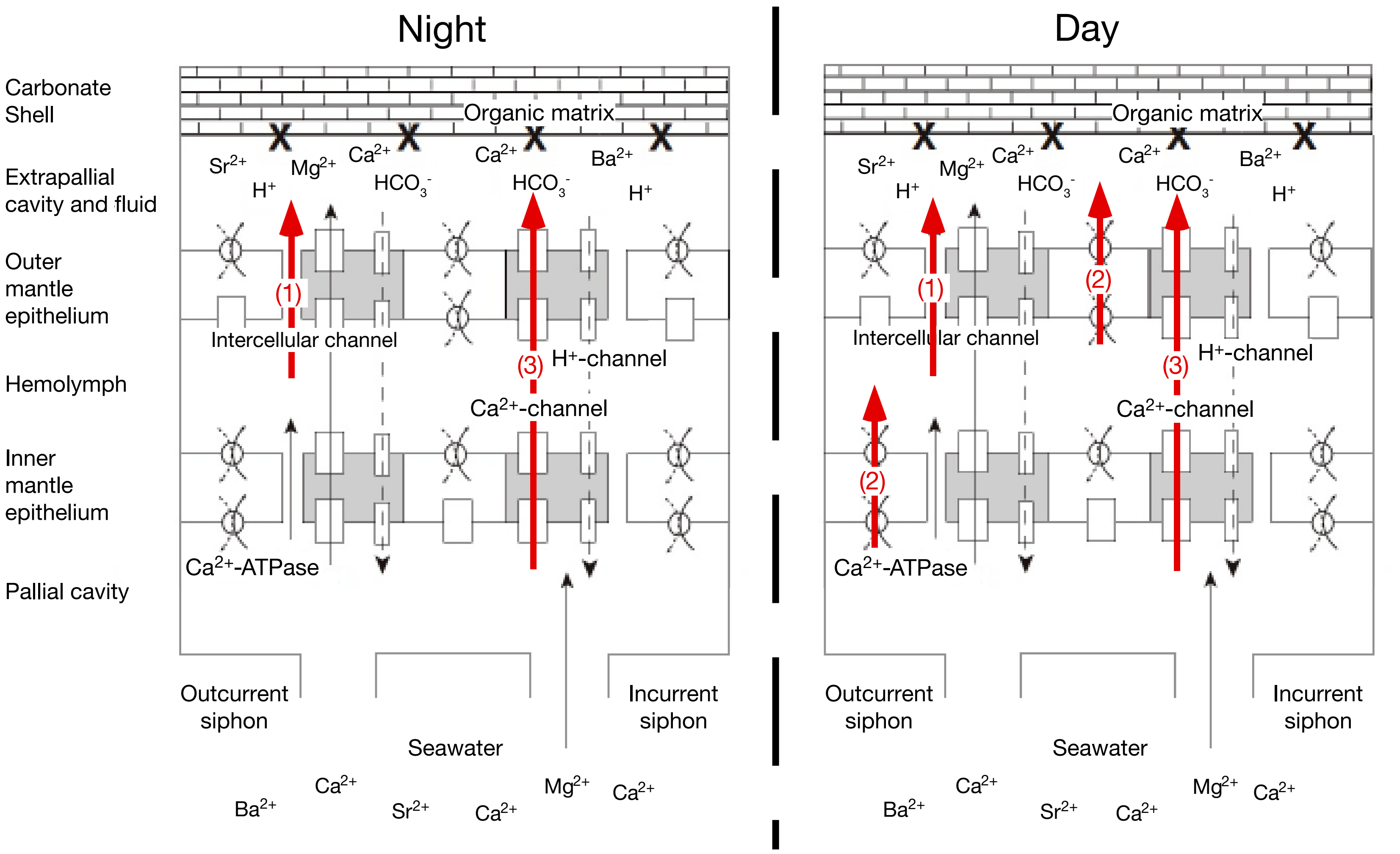

Supplementary Figure S5. Schematic diagram of ion transport pathways in marine bivalve modified from literatures[2,3]. Ca^{2+}-ATPase pump does not work in night time (left side).

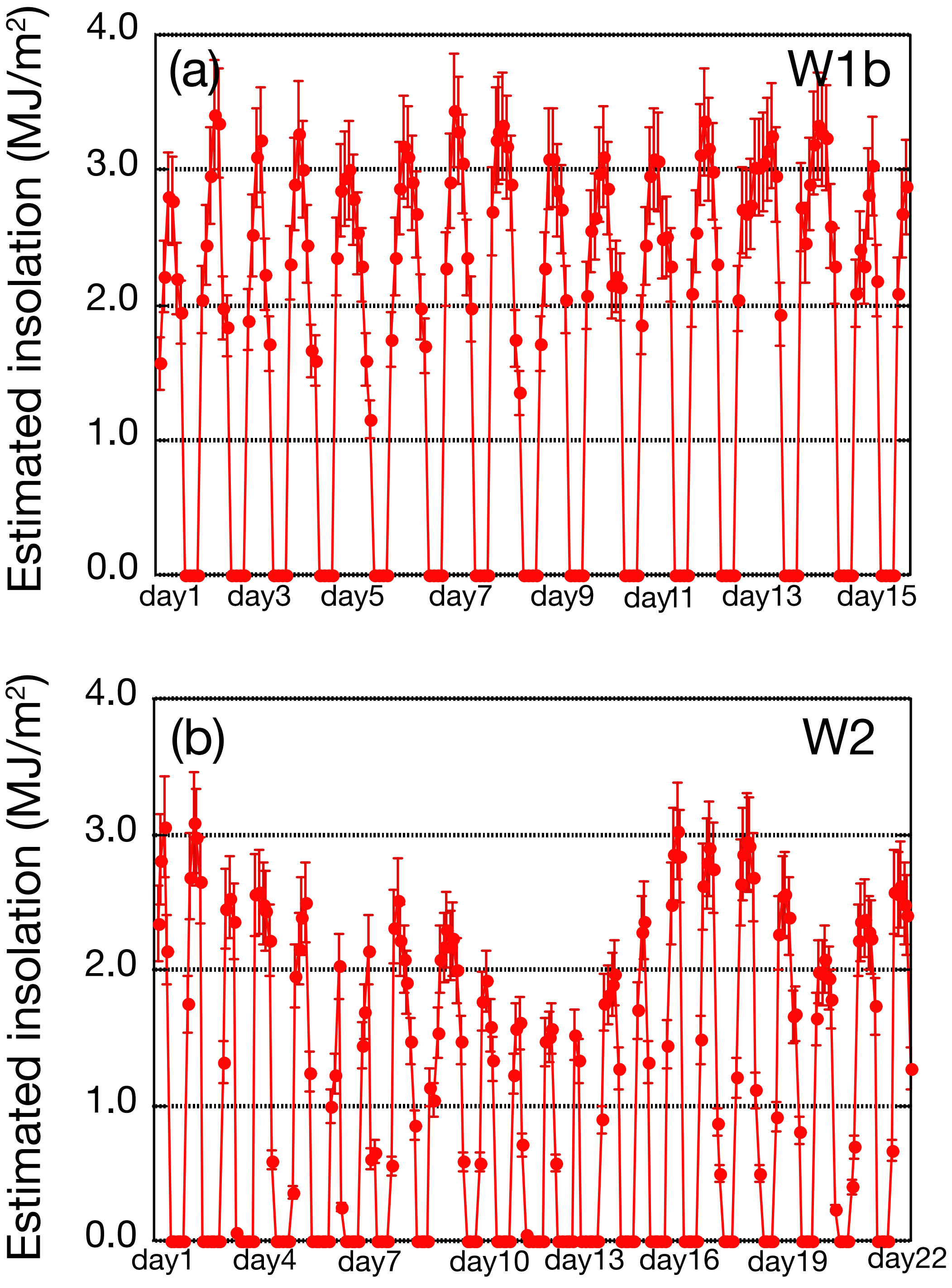

Supplementary Figure S6. Reconstructed hourly insolation of winter time of the middle Holocene by high resolution analysis of Sr/Ca ratio. (a) On the section of W1b in Fig. 1b and (b) that of W2.

Supplementary References

1. Sano, Y. et al. Past daily light cycle recorded in the strontium/calcium ratios of giant clam shells. *Nature Commun.* **3,** 761 (2012).
2. Carré et al. Calcification rate influence on trace element concentrations in aragonitic bivalve shells: Evidences and mechanisms. *Geochim. Cosmochim. Acta* **70,** 4906-4920 (2006).
3. Hippler et al. Exploring the calcium isotope signature of *Arctica islandica* as an environmental proxy using laboratory- and field-cultured specimens. *Palaeogeogr. Palaeocl.* **373,** 75-87 (2013).
